# The psychosocial factors in the formation of symptoms of dementia

**DOI:** 10.1192/j.eurpsy.2021.351

**Published:** 2021-08-13

**Authors:** A. Sidenkova

**Affiliations:** Psychiatry, Ural State Medical University, Yekaterinburg, Russian Federation

**Keywords:** PSYCHOSOCIAL FACTORS, dementia, noncognitive SYMPTOMS OF DEMENTIA

## Abstract

**Introduction:**

The growing prevalence of severe cognitive impairment in populations, the involvement of a significant number of people of working age in the medical, psychological and social problems associated with dementia, the insufficiency and inconsistency of information about the mechanisms of formation of these disorders actualize a comprehensive social study of dementia.

**Objectives:**

the psychosocial mechanisms of the formation of clinical, functional disorders in dementia, to develop comprehensive medical and psychosocial programs to help patients with dementia and those involved in caring for them, based on the proposals of the psychosocial model of dementia

**Methods:**

A selective observational comparative dynamic study of 315 people with Alzheimer’s dementia and 214 people who care for the patients was carried out.

**Results:**

Changes in family-role and social parameters, a high level of “expressed” emotions of caregivers have an adverse effect on the development of psychotic (r = 0.618), affective (r = 0.701), behavioral (r = 0.837) dementia disorders. The degree of adherence to anti-dementia therapy by the caregiver is one of the important factors determining the amount of care received by the patient (r = 0.698). Agitation / aggression (r = 0.761), anxiety (r = 0.562), sleep disturbances (r = 0.521) contribute to increased compliance. The low satisfaction of the caregiver with premorbid (r = 0.698) and current (r = 0.653) relationships with the patient leads to a decrease in the compliance of the caregiver.

**Conclusions:**

The mechanism of psychopathological symptoms, functional disorders is heterogeneous, depending on the biological causes and psychosocial conditions of functioning of patients.

**Disclosure:**

No significant relationships.

